# Gene expression parallels synaptic excitability and plasticity changes in Alzheimer’s disease

**DOI:** 10.3389/fncel.2015.00318

**Published:** 2015-08-25

**Authors:** Carlos A. Saura, Arnaldo Parra-Damas, Lilian Enriquez-Barreto

**Affiliations:** Institut de Neurociències, Departament de Bioquímica i Biologia Molecular, Centro de Investigación Biomédica en Red Enfermedades Neurodegenerativas (CIBERNED), Universitat Autònoma de BarcelonaBarcelona, Spain

**Keywords:** Alzheimer’s disease, memory, transcriptome, neurodegeneration, Aβ, gene expression

## Abstract

Alzheimer’s disease (AD) is a neurodegenerative disorder characterized by abnormal accumulation of β-amyloid and tau and synapse dysfunction in memory-related neural circuits. Pathological and functional changes in the medial temporal lobe, a region essential for explicit memory encoding, contribute to cognitive decline in AD. Surprisingly, functional imaging studies show increased activity of the hippocampus and associated cortical regions during memory tasks in presymptomatic and early AD stages, whereas brain activity declines as the disease progresses. These findings suggest an emerging scenario where early pathogenic events might increase neuronal excitability leading to enhanced brain activity before clinical manifestations of the disease, a stage that is followed by decreased brain activity as neurodegeneration progresses. The mechanisms linking pathology with synaptic excitability and plasticity changes leading to memory loss in AD remain largely unclear. Recent studies suggest that increased brain activity parallels enhanced expression of genes involved in synaptic transmission and plasticity in preclinical stages, whereas expression of synaptic and activity-dependent genes are reduced by the onset of pathological and cognitive symptoms. Here, we review recent evidences indicating a relationship between transcriptional deregulation of synaptic genes and neuronal activity and memory loss in AD and mouse models. These findings provide the basis for potential clinical applications of memory-related transcriptional programs and their regulatory mechanisms as novel biomarkers and therapeutic targets to restore brain function in AD and other cognitive disorders.

## Introduction

The rise of life expectancy has profoundly increased the aging population, and hence the prevalence of age-related cognitive disorders, including Alzheimer’s disease (AD). AD is a devastating neurological disorder characterized by early episodic memory deficits that progresses with cognitive impairments and neuropsychiatric symptoms and finally ends with general disabling dementia. The disease is preceded by a presymptomatic or preclinical stage that can last for years during which the clinical symptoms do not manifest but the pathological process starts (Sperling et al., 2014). In a subsequent prodromal stage named mild cognitive impairment (MCI), the disease is characterized by impairment of memory (amnesia) and other cognitive functions. The majority of subjects with MCI, which represent 10–20% of population older than 65 years, suffer from the disease in the following years (Petersen, 2011).

Memory decline is accompanied by pathological features in the brain of AD patients, including accumulation of extracellular amyloid plaques composed of β-amyloid (Aβ) peptides and intracellular neurofibrillary tangles (NFTs) formed by aggregated hyperphosphorylated microtubule-associated protein tau. These pathological lesions accumulate in brain regions essential for memory encoding and storage, such as the medial temporal lobe (MTL) and related cortical areas (Spires-Jones and Hyman, 2014). Tau pathology starts in the entorhinal cortex (EC) and then spreads to the hippocampal formation and limbic and association cortices (Braak and Braak, 1991). Progression of NFTs correlates well with cognitive decline and neuron loss, whereas amyloid plaques are abundant in neocortical regions but they do not correlate with the degree of memory loss (Arriagada et al., 1992; Gomez-Isla et al., 1997).

It is becoming clear that specific memory circuits are affected by changes in synaptic function and plasticity during the course of the disease. Indeed, synapse dysfunction and loss is an early pathological feature that correlates closely with cognitive impairment (Terry, 2000; Scheff et al., 2007). Recent functional imaging studies reveal decreased activity of the MTL in AD patients, whereas function of cortical and temporal lobe regions, particularly the hippocampus, are increased during memory tasks in preclinical and early stages of the disease (i.e., MCI; for review see Sperling et al., 2010). This enhancement of brain activity may represent a compensatory mechanism resulting from reduced neuronal connectivity that can maintain memory encoding at the beginning of the disease process. Notably, enhanced neuronal activity parallels increased expression of genes involved in synaptic transmission and plasticity at presymptomatic or very early AD stages, whereas deregulation of synaptic gene programs occurs at early and late pathological stages. Here, we summarize pathological as well as functional features occurring in the brain of human and AD mouse models during aging, and discuss recent evidences suggesting a relationship between gene expression changes and neuronal activity and memory disturbances during the progression of AD.

## Hippocampal Pathology and Activity in AD

**FIGURE 1:**
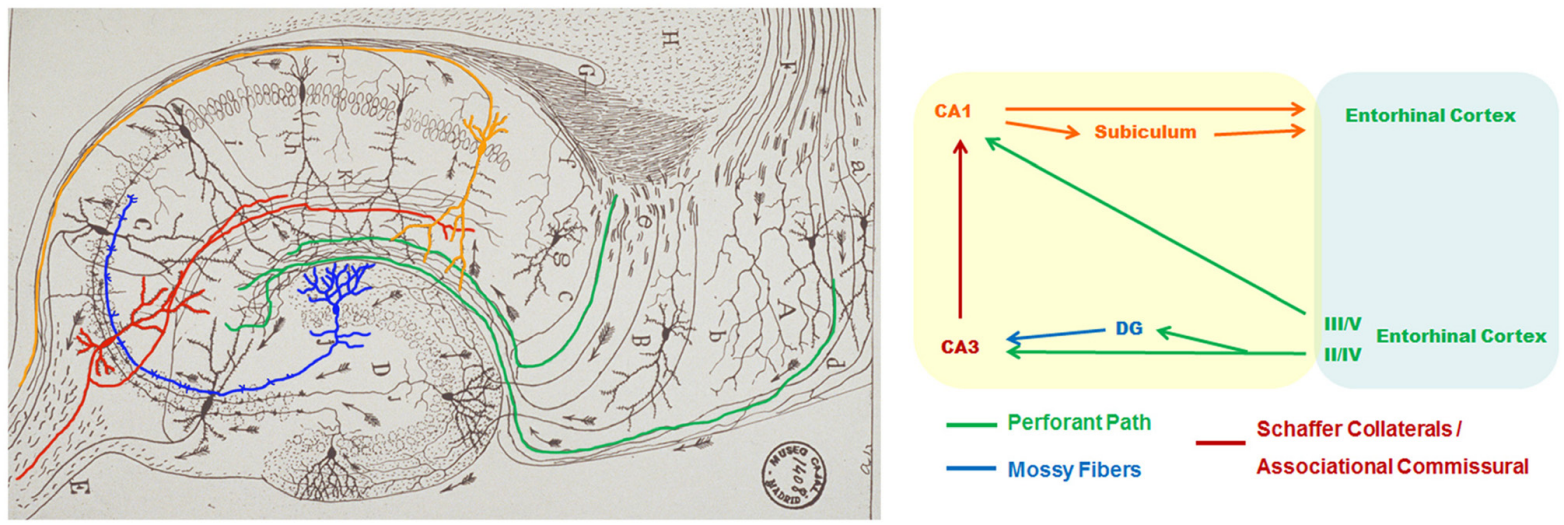
**Hippocampal circuitry in the mouse brain.** Modified original drawing of Santiago Ramón y Cajal’s (1911, left) and schematic diagram (right) of the rodent hippocampal circuitry. The picture shows the flux of excitatory projections (arrows) from entorhinal cortex (EC) neurons (green) directly to CA1 (orange) or CA3 (red) hippocampal pyramidal neurons or indirectly through projections to the dentate gyrus (DG, blue) through the perforant pathway. DG granule neurons project along the mossy fibers to CA3 pyramidal neurons. CA3 axons project through the Schaffer collaterals to CA1 pyramidal neurons, which finally project to the subiculum and deep EC IV- VI layers.

Declarative episodic memories of live facts and events depend on the MTL and connected cortical regions. The MTL includes the hippocampal formation (CA fields, dentate gyrus and subiculum), amygdala and adjacent cortical regions (entorhinal, perirhinal, and parahippocampal cortices; Squirre and Zola-Morgan, 1991). The EC receives cortical sensory information and projects excitatory inputs directly to CA1 pyramidal neurons or to the dentate gyrus and CA3 hippocampus through the perforant pathway (Van Hoesen and Pandya, 1975). CA3 neurons project Schaffer collaterals to CA1 pyramidal neurons, which finally project to the subiculum and deep EC layers IV, V and VI (EC-IV-VI; **Figure 1**). The MTL undergoes atrophy and hypometabolism not only in AD but also in MCI stages (Press et al., 1989; Mosconi et al., 2005; La Joie et al., 2013), an effect observed at least 4 years in advance to cognitive symptoms (Tondelli et al., 2012). Indeed, disruption of the hippocampus, a critical component of this memory circuit, is sufficient to produce anterograde amnesia (Zola-Morgan et al., 1986).

The EC is severely affected by pathological events and neurodegeneration early in AD, likely contributing to memory impairment. AD brains are characterized by a specific pattern of degenerating neurons in EC-II/IV layers and subiculum (Hyman et al., 1984). Cholinergic neurons are particularly vulnerable in AD, and therefore classical therapeutic treatments are based on acetylcholinesterase inhibition. One of the earliest pathological features linked to AD progression is accumulation of NFTs, which occurrs in at least 70% of brains of healthy individuals at sixties (Nelson et al., 2012). Tau pathology starts in the EC and continues to CA1/subiculum field and amygdala prior to clinical symptoms (Braak stages I–II; Hyman et al., 1986; Arriagada et al., 1992). As AD progresses, tau pathology propagates in a sequential regional fashion to limbic and association cortices (Braak stages III–VI) apparently through an aggregation spreading mechanism (Braak and Braak, 1991; Clavaguera et al., 2013). Indeed, NFTs and amyloid plaques are abundant in the terminal sites of the EC projections such as the dentate gyrus (Hyman et al., 1990). Collectively, progressive accumulation and spreading of pathological hallmarks in the MTL suggests that disruption of this neural circuit may contribute to memory decline during the progression of the disease.

Functional magnetic resonance imaging (fMRI) studies show decreased activity and connectivity of the hippocampus, and temporal and prefrontal cortices during episodic memory tasks in AD patients [(Press et al., 1989; Small et al., 1999; Sperling et al., 2003; Pariente et al., 2005; Bai et al., 2009), for review see (Dickerson and Sperling, 2009)]. By contrast, MCI subjects show abnormal activation of the hippocampus and EC during face-name, visual object and verbal associative memory tasks (Dickerson et al., 2005; Hamalainen et al., 2007; Kircher et al., 2007). Compared with healthy aged controls, asymptomatic subjects at risk for AD, including *presenilin-1* (*PSEN1*) C410Y and E280A carriers, show higher activation of the hippocampus and frontal and temporal cortices during associative memory encoding years before clinical symptoms (Bassett et al., 2006; Mondadori et al., 2006; Yassa et al., 2008; Reiman et al., 2012). This increase of brain activity seems to reflect a compensatory mechanism to overcome neural dysfunction in preclinical stages, a process that may be necessary for appropriate memory encoding and retrieval (Kircher et al., 2007; O’Brien et al., 2010; Sperling et al., 2010). Taken together, these studies demonstrate increased activity of the MTL early during presymptomatic AD stages followed by decreased activity as the disease progresses.

## Cortical Default Network in AD

Memory encoding and retrieval are affected by interconnected neocortical regions known as the default mode network, which is active at wakeful rest and deactivates during memory encoding (Buckner et al., 2008). The default mode network is connected to the MTL and includes the medial prefrontal cortex, the posteromedial cingulate cortex, the adjacent ventral precuneus, and the medial, lateral and inferior parietal cortices (Kobayashi and Amaral, 2007).

Specific regions of the cortical default network are functionally disrupted in AD and subjects at risk for AD (i.e., MCI; for a review, see Sperling et al., 2010). Hyperactivation of parietal and prefrontal cortices during memory encoding is accompanied by hippocampal hypoactivation in AD patients (Grady et al., 2003; Pariente et al., 2005). Reduced default network connectivity at rest, but increased activity during performance of attentional and associative memory tasks is observed in MCI and mild AD stages (Koch et al., 2014). Similar to AD patients, older cognitively normal subjects with brain amyloid deposition show significant reduced connectivity of the default network to the hippocampus in resting state (Hedden et al., 2009; Sheline et al., 2010). More striking, asymptomatic subjects with AD-linked autosomal dominant *PSEN1* mutations show decreased activity of the precuneus/posterior cingulate and parietal cortex in resting state (Chhatwal et al., 2013), but increased activity of the frontal, parietal and prefrontal cortex during memory encoding (Wishart et al., 2006; Reiman et al., 2012). These results indicate disruption of the default mode network years before cognitive or behavioral symptoms, which suggests that early AD-associated pathology exerts deleterious functional effects on distinct memory circuits prior to memory impairment.

## Effect of Aβ on Hippocampal Activity and Memory in AD Mouse Models

Mice expressing human *β-amyloid precursor protein* (*APP), TAU* and/or *PSEN1* genes harboring familial AD-linked mutations develop AD pathological hallmarks, neuroinflamation and memory impairments (McGowan et al., 2006). APP and APP/PS1 transgenic mice develop age-dependent amyloid deposits and memory impairments in the absence of tau inclusions (Hsiao et al., 1996; Chapman et al., 1999; Koistinaho et al., 2001). APP transgenic mice show spatial and contextual memory impairments tightly associated with changes in long-term potentiation (LTP), a form of synaptic plasticity thought to be the cellular basis of learning and memory. Tg2576 (Swedish: APP KM670/671NL), APP_Sw,Ind_ (J20) and APP V717I transgenic mice that develop amyloid plaques display impaired hippocampal synaptic plasticity and memory deficits (Chapman et al., 1999; Dewachter et al., 2002; Saura et al., 2005). In APP transgenic mice, including PDAPP (Indiana: APP V717F), Tg2576, APPLd2 (London: V642I), APP23 (Swedish), APP_Sw,Ind_ (J20), APP/PS1 and 3xTg-AD (APP Swedish, Tau P301L, PS1 M146V), altered hippocampal synaptic plasticity and memory deficits precede amyloid plaque pathology suggesting that disruption of memory neural circuits is independent of plaque deposition (Dodart et al., 1999; Hsia et al., 1999; Koistinaho et al., 2001; Kelly et al., 2003; Van Dam et al., 2003; Dominguez-del-Toro et al., 2004; Billings et al., 2005; Saura et al., 2005; Jacobsen et al., 2006; Gruart et al., 2008). Notably, hippocampal-dependent synaptic plasticity and memory deficits in 3xTg-AD, APP_Sw,Ind_ and ArcAβ transgenic mice are associated with the presence of intraneuronal Aβ accumulation, which precedes amyloid plaques (Billings et al., 2005; España et al., 2010a).

**FIGURE 2:**
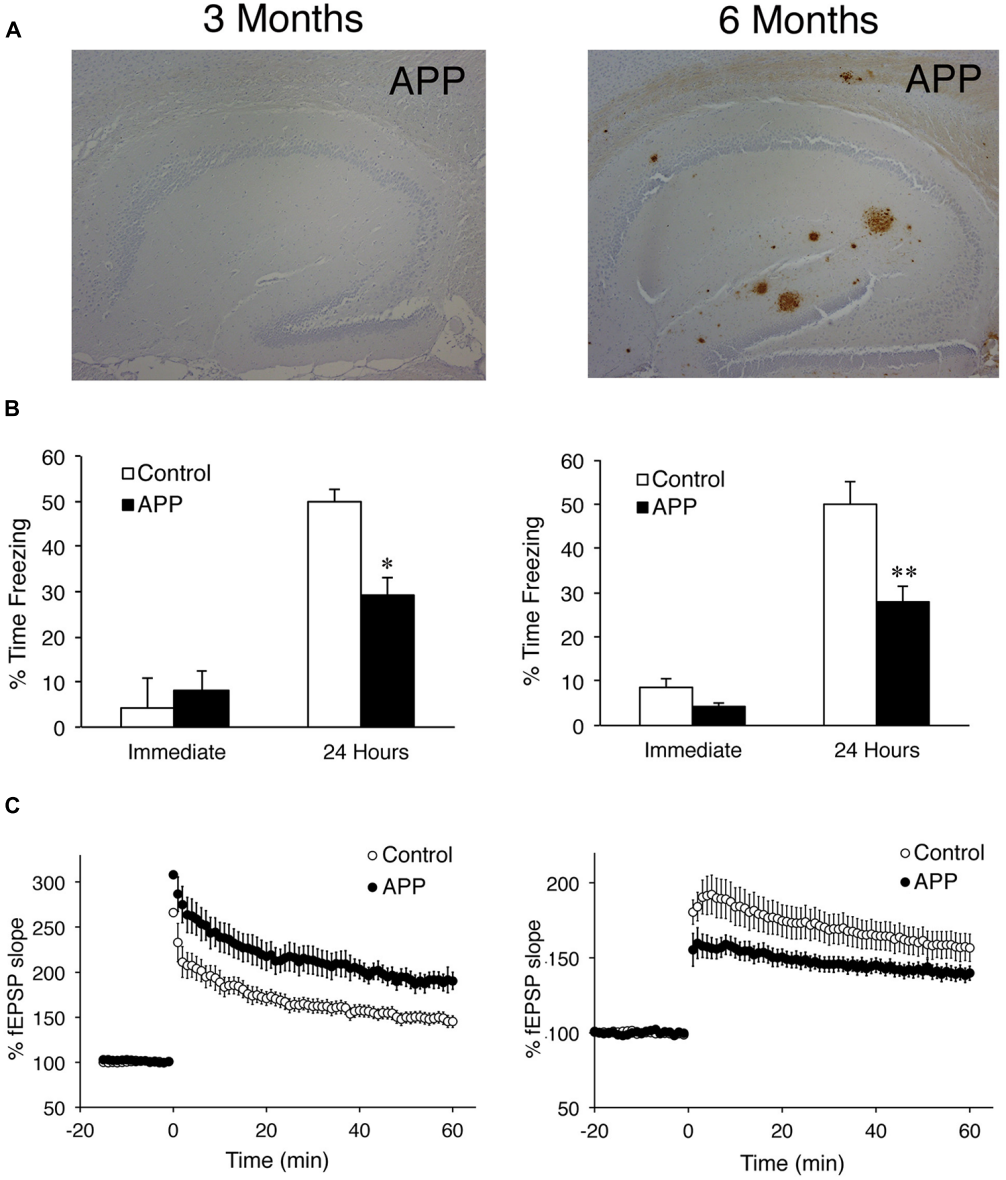
**Age-dependent pathological, synaptic plasticity and associative memory changes in APP transgenic mice. (A)** Brain sections of 3–6 months-old APP_Sw,Ind_ (J20) transgenic mice (APP) stained with an anti-Aβ antibody revealing the presence of amyloid plaques in the hippocampus at 6 months. **(B)** Contextual associative memory in one-shock contextual fear conditioning task. Three and six month-old APP mice exhibit significantly reduced levels of freezing at 24 h indicating disruption of long-term associative memory. Data represent the mean ± SEM. ^∗^*P* < 0.05, ^∗∗^*P* < 0.0001. **(C)**, Time course of LTP induction at the CA1 Schaffer collaterals after theta burst stimulation (TBS) in 3- and 6-months old non-transgenic (control) and APP mice (*n* = 5–7). Notice the differential LTP in APP mice at 3 months (up) and 6 months (down) compared to the respective non-transgenic (control) mice. fEPSP, field excitatory postsynaptic potentials. Images are adapted from Saura et al. (2005).

It has been recently postulated that synaptic excitability changes may alter memory networks leading to cognitive disturbances in AD (Santos et al., 2010, for review). APP_Sw,Ind_ (J20) mice show prior to amyloid plaque deposition enhanced synaptic plasticity in the Schaffer collateral pathway coinciding with early hippocampal-dependent memory deficits (Saura et al., 2005). During aging, APP_Sw,Ind_ mice develop associative memory deficits accompanied by amyloid plaque accumulation and LTP impairments in the hippocampus (**Figure 2**). Similarly, young free-plaque TgCRND8 and 3xTg-AD mice show increased synaptic plasticity caused by enhancement of synaptic excitability in the hippocampus, a phenotype associated with episodic memory impairments (Jolas et al., 2002; Davis et al., 2014). Several studies have also shown increased neuronal hyperactivity and excitability in the cortex of young APP transgenic mice before or when the first amyloid plaques appear (Palop et al., 2007; Busche et al., 2008; Minkeviciene et al., 2009; Gurevicius et al., 2013). This increased excitability is likely responsible for spontaneous epileptic seizures and premature death of APP mice (Palop et al., 2007; Minkeviciene et al., 2009). Enhancement of neuronal activity associated with early pathological and memory changes in AD mouse models resembles the clinical symptoms of MCI subjects (see above).

**FIGURE 3:**
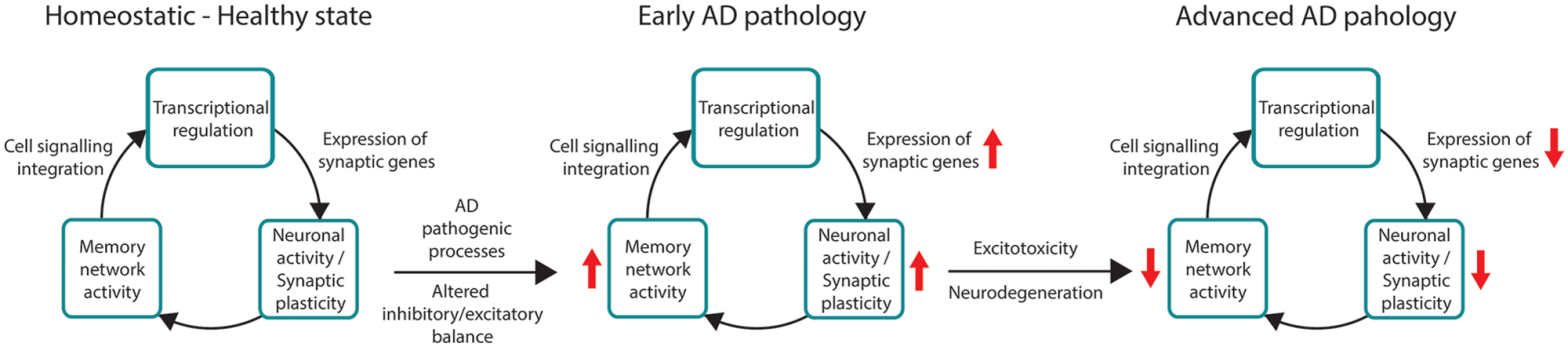
**Hypothetical model linking expression of synaptic genes and neuronal and memory network activities during AD progression.** In healthy state, gene transcription controls expression of synaptic genes to maintain neuronal activity and synaptic plasticity in active memory circuits. In prodromal and very early AD stages, pathological changes increase expression of synaptic genes contributing to inhibitory/excitatory imbalance resulting in enhancement of synaptic excitability and plasticity in memory circuits. At intermediate and severe AD stages, sustained neuronal dysfunction causes transcriptional deregulation of synaptic genes resulting in synapse dysfunction and plasticity impairments, which contributes to memory network disruption and neurodegeneration.

The mechanism by which Aβ induces neuronal hyperexcitability is mediated by an increase of spontaneous action potential firing likely due to an impairment of inhibitory interneuron activity and/or increase of presynaptic vesicle release (Palop et al., 2007; Minkeviciene et al., 2009; Fogel et al., 2014). In this regard, hyperactivity of CA1 hippocampal neurons caused by loss of somatostatin inhibitory interneurons results in memory disturbances in APP transgenic mice (Perez-Cruz et al., 2011). By contrast, increasing the inhibitory activity of parvalbumin interneurons by restoring the voltage-gated sodium channel subunit Nav1.1 improves memory in APP_Sw,Ind_ mice (Verret et al., 2012). Finally, Aβ contributes to emotional psychiatric disturbances by disrupting glutamatergic excitatory/GABAergic inhibitory neurotransmission in the basolateral amygdala (España et al., 2010a). Based on these results, it is plausible that early Aβ accumulation affects the inhibitory/excitatory neuronal balance of specific memory-related neural circuits. This will result in increased neuronal excitability leading to excitotoxicity and synapse and neuronal loss at later pathological stages, when plaque load, synaptic plasticity deficits and memory loss are prominent (**Figure 3**).

## Synapse Dysfunction in AD

Synapse dysfunction is a common pathological feature of several dementing disorders being the major correlate of cognitive impairment in AD (Terry et al., 1991). Synapse loss affects different neuronal populations and neurotransmitter systems in brains of AD subjects (Masliah et al., 1990; Scheff et al., 1990, 2007). Individuals with amnestic MCI and AD have significantly fewer synapses and synaptic proteins in CA1 hippocampus and inferior temporal and posterior cingulate gyrus (Scheff et al., 2007). Accumulation of soluble toxic forms of tau and Aβ at synapses may be a crucial event leading to synapse loss and neurodegeneration (Spires-Jones and Hyman, 2014). Thus, loss of dendritic spines in cortical pyramidal neurons parallels tau phosphorylation during aging (Merino-Serrais et al., 2013), whereas soluble Aβ peptides and oligomers induce synapse loss in mice, rats and non-human primates (Forny-Germano et al., 2014). In APP transgenic, synapse loss and morphology changes are common features that precede amyloid deposition (Lanz et al., 2003; Wu et al., 2004; Rutten et al., 2005; Jacobsen et al., 2006; Wilke et al., 2014). Interestingly, reduced spine density in hippocampal neurons is associated with synaptic plasticity and memory deficits in Tg2576 mice (Jacobsen et al., 2006; Rocher et al., 2008; D’Amelio et al., 2011; Perez-Cruz et al., 2011; Ricobaraza et al., 2012).

The molecular mechanisms leading to synapse dysfunction and loss in AD are largely unclear. Aβ oligomers impair glutamatergic neurotransmission in an activity-dependent manner (Lacor et al., 2004; Deshpande et al., 2009) and cause synapse loss by postsynaptic mechanisms involving deregulation, removal and/or mistargeting of extrasynaptic NMDA and synaptic α-amino-3-hydroxy-5-methylisoxazole-4-propionic acid (AMPA) glutamate receptors (Shankar et al., 2007; D’Amelio et al., 2011; Miñano-Molina et al., 2011). For instance, reduced phosphorylated and surface expression of GluA1 is associated with early spatial memory deficits in APP transgenic mice (Miñano-Molina et al., 2011). Pharmacological treatments that inhibit aberrant extrasynaptic NMDA receptors or activate cAMP/PKA/CREB signaling reverse Aβ-induced dendritic spine loss and memory deficits (Smith et al., 2009; Talantova et al., 2013).

On the other hand, both Aβ and APP modulate excitatory presynaptic vesicle release in an activity-dependent manner (Abramov et al., 2009; Fogel et al., 2014), whereas neuronal activity modulates generation and deposition of Aβ *in vivo* (Bero et al., 2011), suggesting that neuronal hyperactivity can contribute to Aβ generation and accumulation. Taken together, these results point toward a bidirectional regulation between Aβ and neuronal activity through presynaptic and postsynaptic mechanisms.

## Differential Brain Gene Expression in Presymptomatic and Pathological AD Stages

Cognitive decline is associated with changes of gene expression in the brain during aging and AD. Transcriptome profile studies indicate that genes related to synaptic function, energy metabolism and protein synthesis are downregulated in the brain during aging, while expression of inflammatory genes increases especially in the sixth to seventh decades of life (Berchtold et al., 2008; Cribbs et al., 2012; Kumar et al., 2013). Transcription of genes associated with neuron loss, glial activation and lipid metabolism increases with aging while inflammatory cytokines and microglial genes are activated early in AD (Podtelezhnikov et al., 2011), which corroborates an early inflammatory response in AD (Parachikova et al., 2007). Systems biology analysis identified two relevant pathways related to mitochondrial/energy metabolism and synaptic plasticity conserved between AD and aging (Miller et al., 2008). Interestingly, *APP* and *tau* transcripts are upregulated and regulators of APP metabolism (*BACE1, PSEN1, PSEN2*) and tau phosphorylation (*MARK1/3/4, CDK5, PINK1*) are downregulated in memory-related brain regions in individuals with moderate and clinical diagnosis of AD (Liang et al., 2010).

Altered expression of genes related to synapse, energy metabolism and transcriptional regulation processes exacerbate in the brain during the progression of AD pathology contributing likely to cognitive dysfunction (Yao et al., 2003; Blalock et al., 2004; Liang et al., 2008; Miller et al., 2008; Tan et al., 2010; Silva et al., 2012; Berchtold et al., 2013). Remarkably, downregulation of synaptic gene transcripts in CA1 hippocampal neurons of MCI/AD brains correlates with pathological cognitive status (Ginsberg et al., 2012; Counts et al., 2014). By contrast, genes changes related to metabolic/mitochondrial function occur in neurons and astrocytes in AD brain (Liang et al., 2008, 2010; Sekar et al., 2015). In astrocytes, deregulation of genes associated with cytoskeleton, proliferation, apoptosis, and ubiquitin-mediated degradation occur at early Braak stages, while deregulation of intracellular signaling pathways (PI3K/Akt, MAP, insulin) are associated with late pathological stages (Simpson et al., 2011).

Microarrays comparison analysis of synaptic genes in control and AD brains at different ages (20–99 years) revealed significant expression changes in genes regulating vesicle trafficking/release, neurotransmitter receptors, postsynaptic density, cell adhesion and neuromodulation in normal aging and AD, suggesting that similar synaptic genes are vulnerable to aging and AD (Berchtold et al., 2013). Indeed, expression of genes associated with synaptic signaling and structure, protein biosynthesis and mitochondrial/energy metabolism is predominantly increased in the hippocampus, EC and/or temporal gyrus in MCI and decline in AD (Berchtold et al., 2014). Genes that regulate vesicle and synapse function, including those encoding different isoforms of synaptophysin (*SYP*), SNAP25, synapsin (*SYN*), synaptogyrin (*SYNGR1*), synaptobrevin (*VAMP*), synaptotagmin (SYT), syntaxin-1 (*STX1*), synaptopodin (*SYNPO*) and PSD-95 are downregulated in the hippocampus and EC at moderate and severe AD stages (Liang et al., 2010; Ginsberg et al., 2012; Counts et al., 2014). Indeed, altered expression of genes related to synapse function and plasticity correlates better with AD pathology and clinical severity (Gomez Ravetti et al., 2010; Berchtold et al., 2014). **Table 1** summarizes changes of expression of some synaptic genes in AD brain and mouse models. It should be noticed that transcript changes in AD brain could reflect the loss of neurons and synapses in advanced disease stages, a possibility not generally considered in the majority of these studies.

**Table 1:** Summary of expression of synaptic genes in AD and mouse models.

Gene	Gene name	Function	Model	Region	Levels	References
*ARC*	Activity-regulated cytoskeleton-associated protein	Synapse structure	AD APP_Sw,Ind_ APP/PS1 Tg2576/APP_L_	Hip Hip Hip/Cx Hip	Down Down Down Up	Ginsberg et al. (2012), Palop et al. (2005), España et al. (2010a), Parra-Damas et al. (2014), Dickey et al. (2003), Perez-Cruz et al. (2011)
*CHGA*	ChromograninA	Vesicle trafficking/release	AD AD APP_Sw,Ind_	Hip CSF Hip	Down Down Down	Marksteiner et al. (2002), Simonsen et al. (2008), Perrin et al. (2011) Parra-Damas et al. (2014)
*GRIA*	GluA1 GluA2,3,4	Synaptic transmission	BraakII-IV/ AD APP_Sw,Ind_,APPTg AD/3xTg-AD	Hip, EC, MTG Hip/Cx Hip/Cx Hip	Down Down Down Up/Down	Liang et al. (2010), Wakabayashi et al. (1999), Ginsberg et al. (2012), Parra-Damas et al. (2014), Dickey et al. (2003), Cantanelli et al. (2014)
*NEFL*	Neurofilament	Neuron structure	FTD, AD APP_Sw,Ind_ APP/PS1	CSF Hip Hip/Cx	Up Down Down	Sjögren et al. (2000) Parra-Damas et al. (2014) Dickey et al. (2003)
*NRN1*	Neuritin	Neurite	APP_Sw,Ind_	Hip	Down	Parra-Damas et al. (2014)
*NRX1*	Neurexin 1	Synapse structure	MCI AD	EC, SFG Hip, EC	Up Down	Berchtold et al. (2014)
*NR4A1/2*	Nuclear receptor sub 4, 1/2	Synaptic plasticity	AD APP_Sw,Ind_,APPTg	Hip Hip/Cx	Down Down	Dickey et al. (2003), Chu et al. (2006) España et al. (2010b), Parra-Damas et al. (2014)
*RAB*	RAB2,5,7	Vesicle trafficking	MCI/AD APP_Sw,Ind_	Hip Hip	Up Down	Ginsberg et al. (2010) Parra-Damas et al. (2014)
*SCG2*	Secretogranin II	Neurotransmission	AD APP_Sw,Ind_	Hip Hip	Down Down	Marksteiner et al. (2002) Parra-Damas et al. (2014)
SNAP25	Synaptosomal-associated protein 25kDa	Vesicle trafficking	MCI AD 3xTg-AD	EC, Hip, PC EC, Hip, MTG Hip	Up Down Up/Down	Berchtold et al. (2014), Bossers et al. (2010), Liang et al. (2010) Gatta et al. (2014)
*STX*	Syntaxin1A Syntaxin4A Syntaxin 6 Syntaxin 18	Vesicle trafficking/release	MCI/AD AD/APP_Sw,Ind_ MCI AD AD APP_Sw,Ind_	Hip, MTG, EC Hip Hip, PCG Hip Hip, MTG, PC Hip	Down Down Up Down Down Down	Counts et al. (2014), Liang et al. (2010) Ginsberg et al. (2012), Parra-Damas et al. (2014), Berchtold et al. (2014) Liang et al. (2010) Parra-Damas et al. (2014)
*SYT*	SYT1,3,4 SYT6 SYT1,3,5,6,11,12 SYT4	Vesicle trafficking/ release	BraakII/III MCI AD BraakII/IV APP_Sw,Ind_	PC Hip, PCG Hip, EC, PC Hip, EC, MTG Hip	Up Up Down Down Down	Bossers et al. (2010) Berchtold et al. (2014) Ginsberg et al. (2012), Liu et al. (2006) Liang et al. (2010) Parra-Damas et al. (2014)
*VAMP*	VAMP1,2,4 VAMP1,2,3,4	Vesicle trafficking	MCI AD	Hip, PCG Hip, EC, PC	Up/Down Down	Berchtold et al. (2013, 2014), Counts et al. (2014), Liang et al. (2010)

*AD, Alzheimer’s disease; FTD, frontotemporal dementia; MCI, mild cognitive impairment; EC, entorhinal cortex; Hip, hippocampus; MTG, middle temporal gyrus; PC, prefrontal cortex; PCG, post temporal gyrus; SFG, superior frontal gyrus.*

In the prefrontal cortex, transcriptome changes affecting cell signaling, metabolic, inflammation and neurotransmission pathways occur at early pathological stages coinciding with the presence of intraneuronal Aβ (Bossers et al., 2010). Two patterns of gene regulation can be detected: (1) genes related to synaptic function, ATP synthesis and RNA increase in early pathological stages (Braak 0–III) and decline later (Braak IV–VI), and (2) genes related to cell differentiation/proliferation, metal ion binding, antigen processing and transcriptional regulation decrease early and then increase in late Braak stages (Bossers et al., 2010). Synaptic genes upregulated at early pathological stages include potassium voltage-gated channels (*KCNS3, KCNB1, KCNA1*, and *KCNAB1*), GABA receptors (*GABRA1, GABRD, GABRG2*), vesicle exocitosis (*SNAP25, CPLX1, VAMP7, SYT1, SYT3, SYT4, NAPB*, and *SV2C*) and vesicle endocytosis (*clathrin, CLTC, PACSIN1*) proteins.

These above findings indicate a close relationship between transcriptional deregulation and AD-associated neuropathology in memory-related neural circuits. We therefore hypothesize that increased expression of synaptic genes resulting from excitatory/inhibitory imbalance can enhance neural excitability and circuit activity during pre-symptomatic and very early disease stages of AD. In turn, this leads to global gene deregulation, synaptic dysfunction and degeneration and memory loss during the progression of the disease (**Figure 3**).

## Synaptic Gene Expression Changes in AD Mouse Models

Transcriptome profile studies in AD mouse models have revealed altered expression of genes related to mitochondrial function, metabolism, insulin signaling, calcium homeostasis, inflammation, and synaptic plasticity during AD-like pathological progression (see **Table 1**, for synaptic genes). 3xTg-AD mice shows early hippocampal deregulation of genes linked to mitochondrial morphology and function, neuroinflammation, calcium homeostasis, neurotransmission, neuronal loss, and cell cycle (Gatta et al., 2014). 3xTg-AD mice show age-dependent expression changes on AMPA receptor subunits, with marked reduction of *Gria2 and Gria3* in the hippocampus at 12 months. Interestingly, levels of *Gria2, Gria3, and Gria4* transcripts are increased in the hippocampus of young 3xTg-AD animals suggesting a compensatory mechanism against AD-related synaptic dysfunction (Cantanelli et al., 2014). Several synaptic plasticity genes, including *Arc*, early growth response 1 (*Egr1*), *NR2B, Gria1, Homer-1* and *Nr4a1*/*Nur77*, are significantly reduced in the hippocampus of 18 months-old APP/PS1 transgenic mice coinciding with amyloid plaques and memory deficits (Dickey et al., 2003). Interestingly, expression of genes directly implicated in learning/memory and plasticity is increased in the hippocampus of environmental enriched APP/PS1 (Lazarov et al., 2005).

Recently, comparison of different lines of APP and tau transgenic mice revealed that elevation of immune system genes is associated with appearance of amyloid plaques, whereas reduced expression of synaptic genes and increased cell death genes correlate with cortical and hippocampal tau pathology (Matarin et al., 2015). This result agrees with previous reports showing reduced expression of genes related to glutamatergic (*Arc, Gria1, Gria2, Grik4, Psd95*), or GABAergic (*Gad67, Gabarap-11*) neurotransmission and vesicle trafficking (*Syn3, Syb, Synj, Snap29, Syp, Stx4a, Stx7*) in hTau mice (Alldred et al., 2012), and elevation of inflammatory genes in brain regions containing amyloid deposits in APP mice (Dickey et al., 2003; Landel et al., 2014). In summary, transcriptome analysis demonstrates deregulation of common cellular pathways in several AD transgenic mouse models during AD-associated pathology.

## Activity-Dependent Gene Expression and Memory Deficits in AD Mouse Models

Activity-dependent gene expression is a fundamental mechanism mediating structural changes at synapses during memory formation. Cognitive deficits in human and mice are associated with dysregulation of activity-dependent genes and transcription factors (West and Greenberg, 2011). Downregulation of activity-dependent genes involved in synaptic plasticity and memory, including the activity-regulated cytoskeleton-associated protein (*Arc*), *c-fos* and *Bdnf*, are associated with learning and memory deficits in AD and APP transgenic mice (Phillips et al., 1991; Dewachter et al., 2009; España et al., 2010b). Notably, *ARC* transcripts are significantly reduced at early and advanced AD pathological stages (Ginsberg et al., 2012; Parra-Damas et al., 2014). Similarly, *Arc* expression is markedly decreased in the hippocampus and visual cortex of APP transgenic mice after experience and memory training (Palop et al., 2005; Rudinskiy et al., 2012; Parra-Damas et al., 2014). Paradoxicaly, *Arc* is increased in individual cortical neurons close to amyloid plaques and CA1 pyramidal neurons in APP mice, an effect attributed to neuronal hyperactivity caused by decreased synaptic inhibition (Perez-Cruz et al., 2011; Rudinskiy et al., 2012). Despite the established disruption of activity-dependent gene expression in AD, the regulatory transcriptional mechanisms underlying gene changes causing memory loss in this disease are largely unknown. Understanding these mechanisms may offer new opportunities for therapeutic intervention in cognitive disorders.

To discern transcriptional mechanisms related to memory impairment in AD, we recently performed genome-wide transcriptome analyses in naïve and memory trained non-transgenic and APP_Sw,Ind_ (J9) mice. Gene-annotation analysis revealed a gene cluster of 164 transcripts deregulated in the hippocampus of 6 months-old APP_Sw,Ind_ mice compared to non-transgenic mice after memory training. The biological pathways associated with these genes are learning/memory, neurotransmission, synaptic plasticity, glutamatergic and GABAergic neurotransmission, oxidative phosphorylation and AD (Parra-Damas et al., 2014). Coinciding with initial intraneuronal Aβ accumulation and memory deficits, APP_Sw,Ind_ mice show deregulation of a transcriptional program dependent on the cAMP-response element binding protein (CREB)-regulated transcription coactivator-1 (CRTC1), which includes genes involved in neurotransmission (*Scg2, Syt4, Stx4, Stx18, Rab2a, Gria1, Chga*), synaptic plasticity/memory (*Arc, c-fos, Nr4a1, Nr4a2, Bdnf*) and neuritogenesis (*Nefl, Nrn1*) (Parra-Damas et al., 2014) (**Table 1**). This result is consistent with a decline of synaptic gene transcripts coinciding with the presence of intraneuronal Aβ and preceding synapse loss in human prefrontal cortex at intermediate pathological stages (Bossers et al., 2010).

Genetic and pharmacological studies have shown that disruption of CREB signaling mediates synaptic plasticity and memory impairments in AD (Saura and Valero, 2011). Accordingly, CREB activation ameliorates synaptic and memory deficits in APP transgenic mice (Smith et al., 2009; Yiu et al., 2011), whereas CRTC1 gene therapy reverses early transcriptional changes and memory impairments in AD mice (Parra-Damas et al., 2014). In summary, disruption of CREB/CRTC1-dependent transcription underlies early memory deficits whereas its activation ameliorates AD-related synaptic and memory impairments, which provides evidence that targeting this pathway may be therapeutically beneficial in AD.

## Pathogenic and Therapeutic Implications of Gene Deregulation in AD

AD is the most common form of dementia in the aging population but, unfortunately, current therapies are not effective to ameliorate or reverse the clinical symptoms. Classical pharmacological treatments based on inhibition of acetylcholinesterase (e.g., donepezil, rivastigmine) or excitotoxicity (memantine) slow the disease progression but do not prevent or stop the neurodegeneration process. Alternatively, anti-amyloid immunotherapy treatments that efficiently reduce amyloid plaque burden fail to improve cognitive performance in mild-to moderate AD patients (Doody et al., 2014; Salloway et al., 2014). The discouraging failures of anti-amyloid clinical trials have raised doubts about the contribution of Aβ as the initiating factor in AD pathophysiology (Herrup, 2015). Alternatively, several molecular, genetic and cellular events affected by aging, the main risk factor of the disease, may contribute to neuronal dysfunction and degeneration leading to dementia in AD.

**FIGURE 4:**
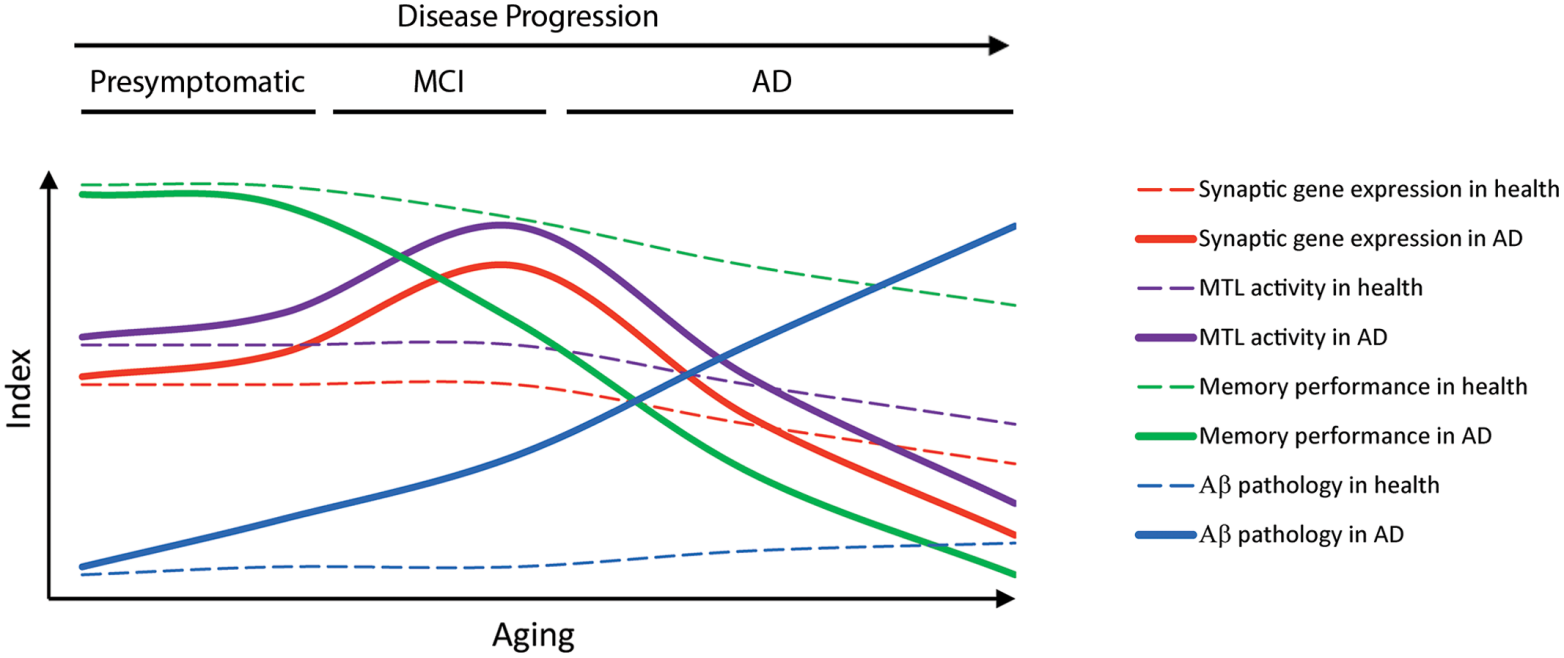
**Temporal progression of pathological and transcriptional changes in aging and AD.** The diagram represents the hypothetical temporal progression of memory deficits, expression of synaptic genes, medial temporal lobe (MTL) activity and Aβ pathology during aging and AD. Enhanced brain activity at prodromal AD stages is associated with increased expression of synaptic genes, whereas decreased brain activity parallels reduction of synaptic genes as the disease progresses.

In this context, deregulation of genes involved in pathological pathways, including oxidative stress, mitochondrial/energy metabolism, synapse dysfunction and inflammation, may be crucial in the etiology of AD. Thus, despite massive gene expression changes in the brain, few vulnerable biological pathways, including energy metabolism, synaptic function/plasticity and inflammation are generally altered in normal aging and AD (Berchtold et al., 2008, 2013; Cribbs et al., 2012). Gene expression deregulation occurs already in presymptomatic or early diseases phases (Bossers et al., 2010; Liang et al., 2010; Berchtold et al., 2014). Thus, synaptic and energy metabolism gene clusters are upregulated early during the disease process declining later at intermediate/severe pathological stages. Based on these results, we hypothesize that upregulation of synaptic genes contributes to increased neural excitability and memory circuit activity at presymptomatic or very early disease stages, which could then trigger gene deregulation, synaptic dysfunction, degeneration and memory loss during the progression of the disease (**Figure 3**). Indeed, changes in expression of synaptic genes parallel altered activity of memory circuits indicating a close relationship between neuropathology, transcriptional deregulation and activity of susceptible memory circuits in AD (**Figure 4**).

How changes of synaptic gene programs contribute to neuronal activity and connectivity alterations leading to memory loss in cognitive disorders is starting to be elucidated. One suggested possibility is that Aβ enhances intrinsic neuronal excitability leading to memory network changes and clinical manifestations of AD (Palop et al., 2007). Therapeutic approaches aimed at reducing neuronal hyperactivity may be beneficial to stabilize synaptic function and improve cognitive abilities at early disease stages. In support of this idea, a recent clinical trial indicates that levetiracetam, an antiepileptic drug that reduces hippocampal hyperactivity, improves cognition in amnestic MCI subjects (Bakker et al., 2012). Levetiracetam treatment also reverses synaptic gene changes as well as functional abnormalities and cognitive deficits in APP transgenic mice (Sanchez et al., 2012), whereas decreasing oxidative stress, excitotoxicity and hyperexcitability without interfering with amyloid or tau pathologies prevents AD-related memory deficits (Isopi et al., 2014). Likewise, the antiepileptic drug valproate ameliorates memory deficits and neuropsychiatric symptoms in APP transgenic mice (España et al., 2010a; Yao et al., 2014), but fails to slow cognitive decline and behavioral symptoms at late diseases stages (Fleisher et al., 2011). The efficacy of anti-hyperactivity drugs for treating AD will ultimately depend on the drug type, dosage and disease stage.

Gene profiling and transcriptional regulatory mechanisms involved in memory loss could offer clinical applications as diagnostic tools, novel biomarkers and therapeutic targets in AD. First, it is conceivable that meta-analysis of transcriptomic data from large population-based cohorts of sporadic AD patients may reveal differentially altered pathways related to specific pathogenic mechanisms, opening new venues to design personalized therapeutic strategies. Second, individual or particular set of genes altered in AD brain could be applied as novel early biomarkers to predict the progression of the disease and to monitor therapeutic effects in personalized medicine. Of relevance, synaptic genes identified by wide-genome profile analysis in mouse models are deregulated in AD brain or biological fluids (**Table 1**). Several of these synaptic proteins, such as BDNF, secretogranin II, synaptotagmin, chromogranin A and SNAP25, have been proposed as novel biomarkers for AD (Simonsen et al., 2008; Li et al., 2009; Perrin et al., 2011; Brinkmalm et al., 2014). Since testing brain tissue could be of limited application in population-based screenings, the use of CSF and blood samples may represent a valuable non-invasive tool for biomarker analysis. Indeed, deregulation of genes and microRNAs (miRNAs) in CSF and/or blood (plasma and blood cells) occurs in AD patients (Chen et al., 2011; Bekris et al., 2013; Roed et al., 2013), and a gene signature in blood related to inflammation, transcription and cell death was recently used for diagnosis and prediction of MCI to AD conversion (Roed et al., 2013). Interestingly, the abundant changes of transcripts of genes related to synaptic plasticity/transmission, neuritogenesis and neurological diseases (AD, Parkinsons’disease, and mental retardation) in blood cells of AD patients suggest a strong link between blood and brain transcriptional profiles in AD (Naughton et al., 2015). Future identification of relevant biomarkers in biological fluids may be useful for early and accurate diagnosis of AD.

An important point is that gene expression is regulated by multiple mechanisms including transcription, translation and posttranscriptional or posttranslational mechanisms. Among these, epigenetic regulation has been the intense focus of research in neurodegenerative diseases in recent years. Epigenetic chromatin remodeling and DNA modifications regulate gene expression during memory formation, whereas epigenetic dysregulation is associated with aging and cognitive disorders (Graff and Mansuy, 2009). Thus, global reduction of DNA methylation and hydroxymethylation occur in the hippocampus of AD patients at early pathological stages (Chouliaras et al., 2013; Sanchez-Mut et al., 2013; De Jager et al., 2014). Other epigenetic factors such as non-coding RNA, in particular miRNAs and long non-coding RNAs, have also received increasing attention in neurodegenerative diseases due to their role in modulating gene expression. A set of miRNAs and long non-coding RNAs are deregulated in brain, blood, and CSF of AD patients (Dorval et al., 2013; Lau et al., 2013), which raises the possibility that non-coding RNAs may play a key role in gene expression deregulation during the course of the disease. Nonetheless, specific miRNAs and long non-coding RNAs affect expression of genes involved in AD pathology including gene regulating APP processing, tau, inflammation and apoptosis (Goodall et al., 2013). The diversity of miRNAs and their potential to target gene expression of multiple pathways offer alternative applications of these molecules as novel biomarkers and therapeutic targets for AD and other neurodegenerative diseases.

Based on the above studies, epigenetic therapeutic approaches have been applied in AD mouse models. As an example, histone deacetylase inhibition increases expression of plasticity genes and ameliorates synaptic pathology and cognitive deficits in APP transgenic mice (Ricobaraza et al., 2012). Long-term systemic treatment with epigenetic drugs may, however, cause broad and deleterious effects on brain function. Alternatively, targeting molecules or pathways regulating specific gene expression programs in vulnerable memory circuits may represent potential therapeutic targets for AD. Interestingly, a recent report demonstrates that a gene therapy approach targeting CRTC1 to enhance expression of specific synaptic genes prevents memory impairments in an AD mouse model (Parra-Damas et al., 2014). A future scientific challenge will be the identification of transcriptome signatures in the brain or biological fluids for early diagnosis and prediction of the disease. In parallel, a better understanding of the expression regulatory mechanisms of genes involved in synaptic dysfunction and neurodegeneration will be crucial to develop efficient therapeutic treatments for AD and other cognitive disorders.

## Conflict of Interest Statement

The authors declare that the research was conducted in the absence of any commercial or financial relationships that could be construed as a potential conflict of interest.
